# Room Temperature Lattice Thermal Conductivity of GeSn Alloys

**DOI:** 10.1021/acsaem.4c00275

**Published:** 2024-05-15

**Authors:** Omar Concepción, Jhonny Tiscareño-Ramírez, Ada Angela Chimienti, Thomas Classen, Agnieszka Anna Corley-Wiciak, Andrea Tomadin, Davide Spirito, Dario Pisignano, Patrizio Graziosi, Zoran Ikonic, Qing Tai Zhao, Detlev Grützmacher, Giovanni Capellini, Stefano Roddaro, Michele Virgilio, Dan Buca

**Affiliations:** † Peter Gruenberg Institute 9 (PGI-9) and JARA-Fundamentals of Future Information Technologies, Forschungszentrum Juelich, Juelich 52428, Germany; ‡ Dipartimento di Fisica, Università di Pisa, Largo Bruno Pontecorvo 3, Pisa 56127, Italy; § IHP - Leibniz Institut für innovative Mikroelektronik, Frankfurt (Oder) 15236, Germany; ∥ CNR − ISMN, Via P. Gobetti 101, Bologna 40129, Italy; ⊥ Pollard Institute, School of Electronic and Electrical Engineering, University of Leeds, Leeds LS2 9JT, United Kingdom; # Dipartimento di Scienze, Università degli Studi Roma Tre, Viale G. Marconi 446, Roma 00146, Italy

**Keywords:** Thermoelectric materials, lattice thermal conductivity, GeSn alloys, CMOS, green computing, energy harvesting

## Abstract

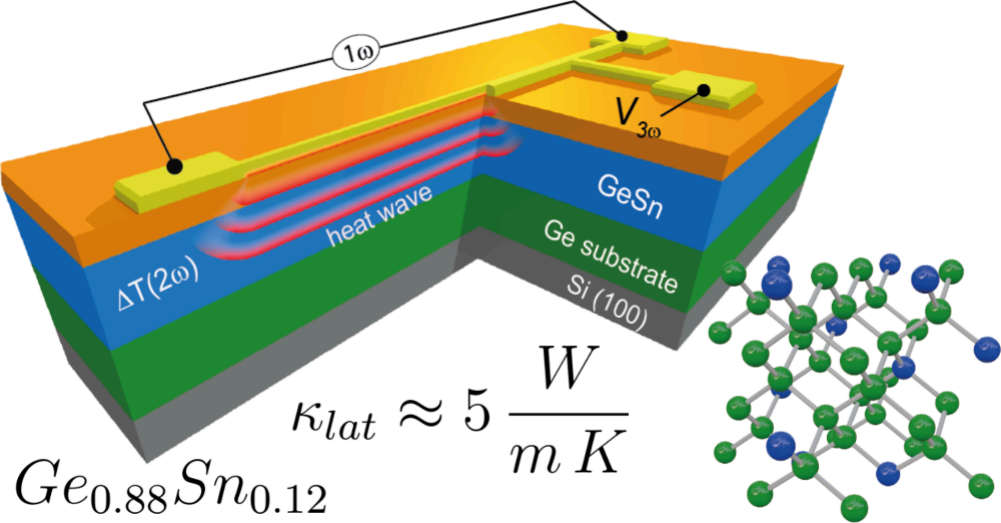

CMOS-compatible materials for efficient energy harvesters at temperatures characteristic for on-chip operation and body temperature are the key ingredients for sustainable green computing and ultralow power Internet of Things applications. In this context, the lattice thermal conductivity (κ) of new group IV semiconductors, namely Ge_1–*x*_Sn_*x*_ alloys, are investigated. Layers featuring Sn contents up to 14 at.% are epitaxially grown by state-of-the-art chemical-vapor deposition on Ge buffered Si wafers. An abrupt decrease of the lattice thermal conductivity (κ) from 55 W/(m·K) for Ge to 4 W/(m·K) for Ge_0.88_Sn_0.12_ alloys is measured electrically by the differential 3ω-method. The thermal conductivity was verified to be independent of the layer thickness for strained relaxed alloys and confirms the Sn dependence observed by optical methods previously. The experimental κ values in conjunction with numerical estimations of the charge transport properties, able to capture the complex physics of this quasi-direct bandgap material system, are used to evaluate the thermoelectric figure of merit *ZT* for n- and p-type GeSn epitaxial layers. The results highlight the high potential of single-crystal GeSn alloys to achieve similar energy harvest capability as already present in SiGe alloys but in the 20 °C–100 °C temperature range where Si-compatible semiconductors are not available. This opens the possibility of monolithically integrated thermoelectric on the CMOS platform.

## Introduction

Waste heat, as a result of industrial processes and human activity, represents a significant opportunity for valorization. While most industries successfully recover high- (>400 °C) and medium-grade (100–400 °C) waste heat, valorizing low-grade waste heat (<100 °C) remains extremely challenging technologically, economically, and is not yet common in practice.^1^ Its conversion into a usable form of energy has a poor thermodynamic efficiency and is difficult to implement. At the same time, the generation of heat is a major bottleneck of Si-based electronics and optoelectronics, especially when high data processing and transmission are considered, and results in the need for external cooling, which in turn, consumes more energy. Consequently, it is becoming necessary to develop new material platforms that could be adopted in the manufacturing of Si-integrated optoelectronic devices, enabling the recovery/saving of energy consumption via built-in energy harvesters.

At the European level, around 1.2 EJ/year of low-temperature heat is available from urban sources such as data centers, metro stations, wastewater treatment plants, and service sector buildings.^2^ Thermoelectric (TE) materials appear to be an ideal solution to fulfill this task since they can provide electrical power gain and/or cooling of critical devices.^3^ However, TE features a poor-to-inexistent presence in integrated circuit consumer products, where temperatures below 100 °C are typical. This is due to one major shortfall: there are no Si-compatible semiconductors with TE capabilities at typical integrated circuit (IC) working temperatures. As such, their development could make a significant contribution to the reduction of IC energy consumption.

The major drawback for thermoelectrics with Si and Ge elemental semiconductors, the workhorses of Si-electronics and Si-photonics, is their large lattice thermal conductivity of 160 W/(m·K) and 60 W/(m·K), respectively, resulting in a poor thermoelectric figure of merit and, in the best case of their SiGe alloys, high-temperature (above 800 °C) TE operation.^4,5^ Recently, the GeSn alloy, a new artificially developed group IV semiconductor,^6−8^ which proved to be extremely promising for monolithic integrated Si-based optoelectronics, has been proposed as a thermoelectric material.^9,10^ Alloying atoms with a larger mass difference is a successful strategy to improve the TE performance as the large mass fluctuation generates a strong scattering of phonons, thus suppressing the lattice thermal conductivity. However, the alloying atoms should best have a small difference of radii to reduce the perturbations induced in the periodic potential and not deteriorate the charge carrier transport, which is also a fundamental requirement for a good TE material. Both these conditions are fulfilled in the GeSn material system since the Sn atoms have a mass 70% higher than that of Ge atoms, while there is only an 18% atomic radius difference between them. Consequently, the incorporation of only a few percent of Sn into the Ge lattice can decrease the Ge host lattice thermal conductivity by more than 1 order of magnitude, to values 200 times lower than that of Si.^9,10^ If one also considers the good electrical conductivity typical for Si-group semiconductors, this may potentially lead to good thermoelectric figure of merit and power factors in the low-temperature range below 100 °C, required for electronic chips or wearable energy harvester electronics.^11,12^

The group IV elements, Ge and Sn, are already intensely studied for thermoelectric, but usually in combination with group VI elements like tellurium and selenium, e.g. SnSe, GeTe, GeSe,^13−15^ which cannot be integrated into a Si foundry. On the contrary, the success in epitaxial growth of GeSn alloys with high crystalline quality already led to the development of advanced vertical nanowires field effect transistors,^16−18^ CMOS invertors,^14^ optically pumped laser,^19−22^ electrically pumped laser^23,24^ or the fabrication of efficient photodetectors and infrared imagers.^25^ Nevertheless, there are only a few scattered investigations on the thermoelectric properties of the GeSn material system, both experimentally and theoretically.^10,26−28^

As a step in this direction, in this work, one major thermoelectric parameter, namely the lattice thermal conductivity κ_latt_, is investigated for a relatively large set of GeSn/Ge/Si epitaxial heterostructures featuring Sn contents up to 14 at.%. The emphasis is here put on the electrical experimental technique, i.e., the so-called “3ω-method” which is then compared with an alternative optical method based on micro-Raman thermometry. The obtained values, phenomenologically parametrized as a function of Sn content and layer thickness, are then used to numerically predict the relevant thermoelectric figures of merit for n-type doped GeSn alloys in the temperature range of interest, <100 °C. For this purpose, a multivalley-bipolar Boltzmann transport model is used. The results highlight the potential of GeSn alloys as a thermoelectric material for energy conversion at low-grade heat and body temperature.

## Material and Devices

**Figure 1:**
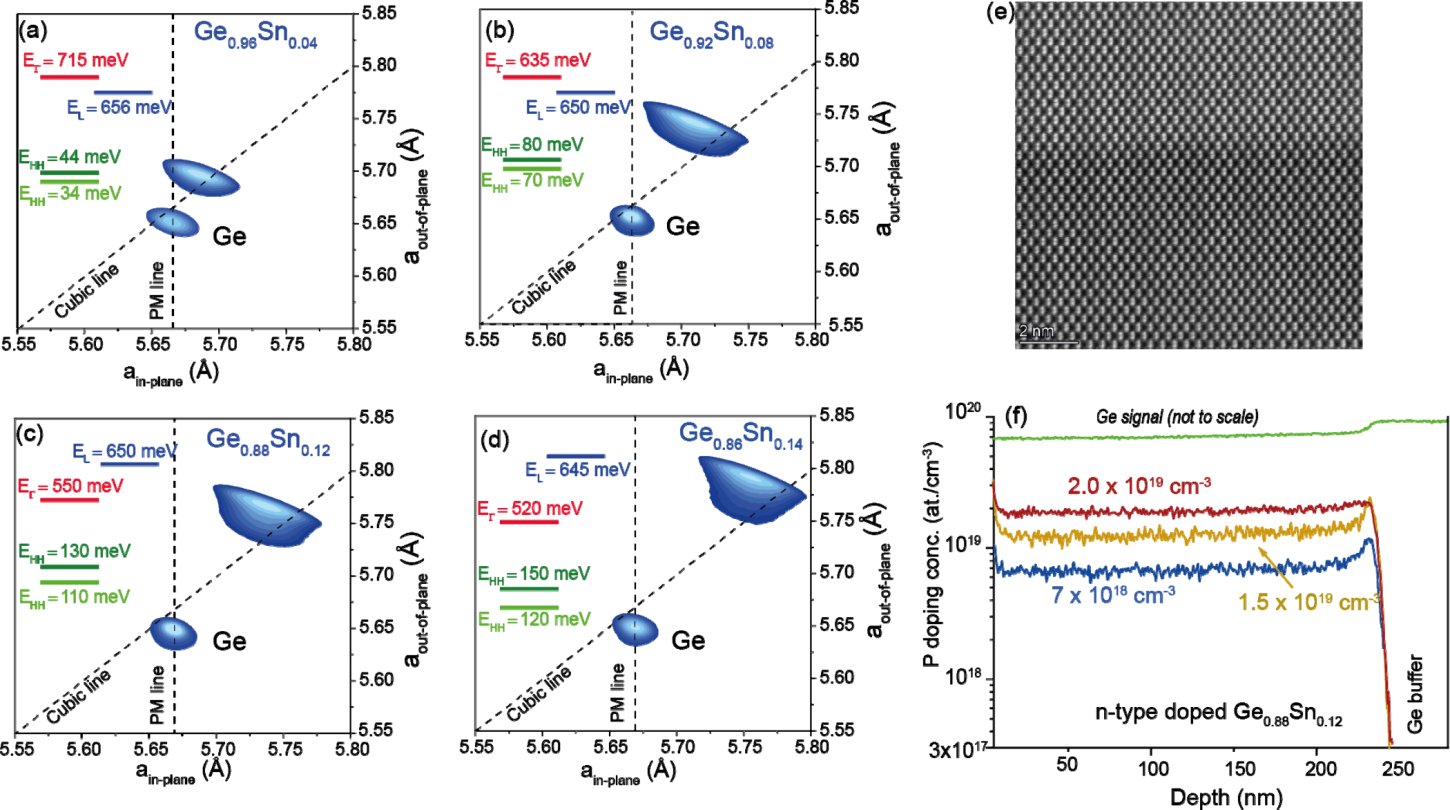
GeSn material: (a–d) Asymmetric XRD-RSM scans of GeSn layers with different Sn content as used in this work along the (224) crystal plane, indicating the shift of GeSn peak toward higher lattice parameters. The cubic line and the pseudomorphic one (PM) are indicated by dashed lines. The electronic band energies are also given. (e) HR-TEM micrograph of a 300 nm Ge_0.88_Sn_0.12_ layer evidencing the high crystalline quality of the layer. (f) SIMS profile of n-GeSn layers doped with different concentrations of phosphorus, showing a uniform doping level throughout the full layer.

The GeSn samples were grown on prime-grade 200 mm Si(100) wafers in an industry-compatible reduced-pressure chemical vapor deposition reactor equipped with showerhead technology that ensures uniform gas precursor distribution over the whole wafer. Due to the large lattice mismatch between Sn and Si, a Ge buffer layer was grown prior to the GeSn deposition to improve the crystal quality. Details on the epitaxy can be found elsewhere.^17,29^ The Sn content and the layer thickness were extracted by fitting the Rutherford backscattering spectra^7,8,30^ (not shown). The layer thickness of GeSn alloys of different stoichiometries was between 250 and 300 nm, except for the 12 at.% Sn, where a set of thicknesses, between 50 and 700 nm, was grown. A set of asymmetric (224) X-ray diffraction reciprocal space mappings (XRD-RSM) corresponding to GeSn layers with Sn concentration between 4 at.% and 14 at.% are shown in Figure 1a–d as a function of the real space in-plane and out-of-plane lattice parameters. The GeSn layer peak position shifts to larger lattice parameters as a result of the incorporation of Sn atoms into the Ge lattice. The Ge peak is below the cubic line, meaning that it is slightly tensile strained, typically +0.15% for thermally treated Ge on Si(100), while the GeSn peaks are above the cubic line, indicating biaxially compressively strained material. The residual in-plane compressive strain is caused by incomplete stress relaxation and amounts to −0.1% for the 4 at.% Sn layer and reaches −0.3% for the 12 and 14 at.% Sn compositions. The energies of the electronic bands of the Ge_1–*x*_Sn_*x*_/Ge buffer including the lattice strain were calculated using the methodology given in Supporting Information of ref. (21), and are given as insets in Figures 1a–d. With increasing the Sn content, the energy of the Γ-valleys decreases faster than the L-valley energy leading to a transition from indirect to direct semiconductor,^31,32^ as seen in Figures 1c,d for the cases of 12 at.% and 14 at.% Sn. The high crystalline quality of the epitaxial layers is evidenced in Figure 1e where a high-resolution transmission electron micrograph (HR-TEM) taken in the middle of the Ge_0.88_Sn_0.12_ layer is shown.

The GeSn layers are not intentionally doped, but the presence of crystalline point defects, typically vacancies, linked to the plastic relaxation of the heteroepitaxial strain led to an electrical p-type behavior with an acceptor-like state density estimated to be lower than 1–5 × 10^17^ cm^–3^. For low doping levels, the calculated carrier thermal conductivity^33^ κ_el_, is <0.01 W/m·K, and thus has a negligible contribution to the total thermal conductivity κ_GeSn_*= κ*_latt_*+ κ*_el_. Consequently, or in the case of unintentional doped GeSn layers, the measured thermal conductivity values κ_GeSn_, are regarded as the lattice thermal conductivity κ_latt_. To address the role of doping on the TE properties, a set of samples was grown under identical conditions but with different flows of the PH_3_ gas precursor flux. This codoping process resulted in phosphorus concentrations in the 1 × 10^18^ to 2 × 10^19^ at/cm^3^ range with a uniform spatial distribution through the layer as measured by secondary ion mass spectrometry (SIMS) (see Figure 1f). For such doping levels, the κ_el_ is becoming larger and cannot be neglected. The experimentally measured thermal conductivity is regarded as total thermal conductivity κ_GeSn_.

**Figure 2:**
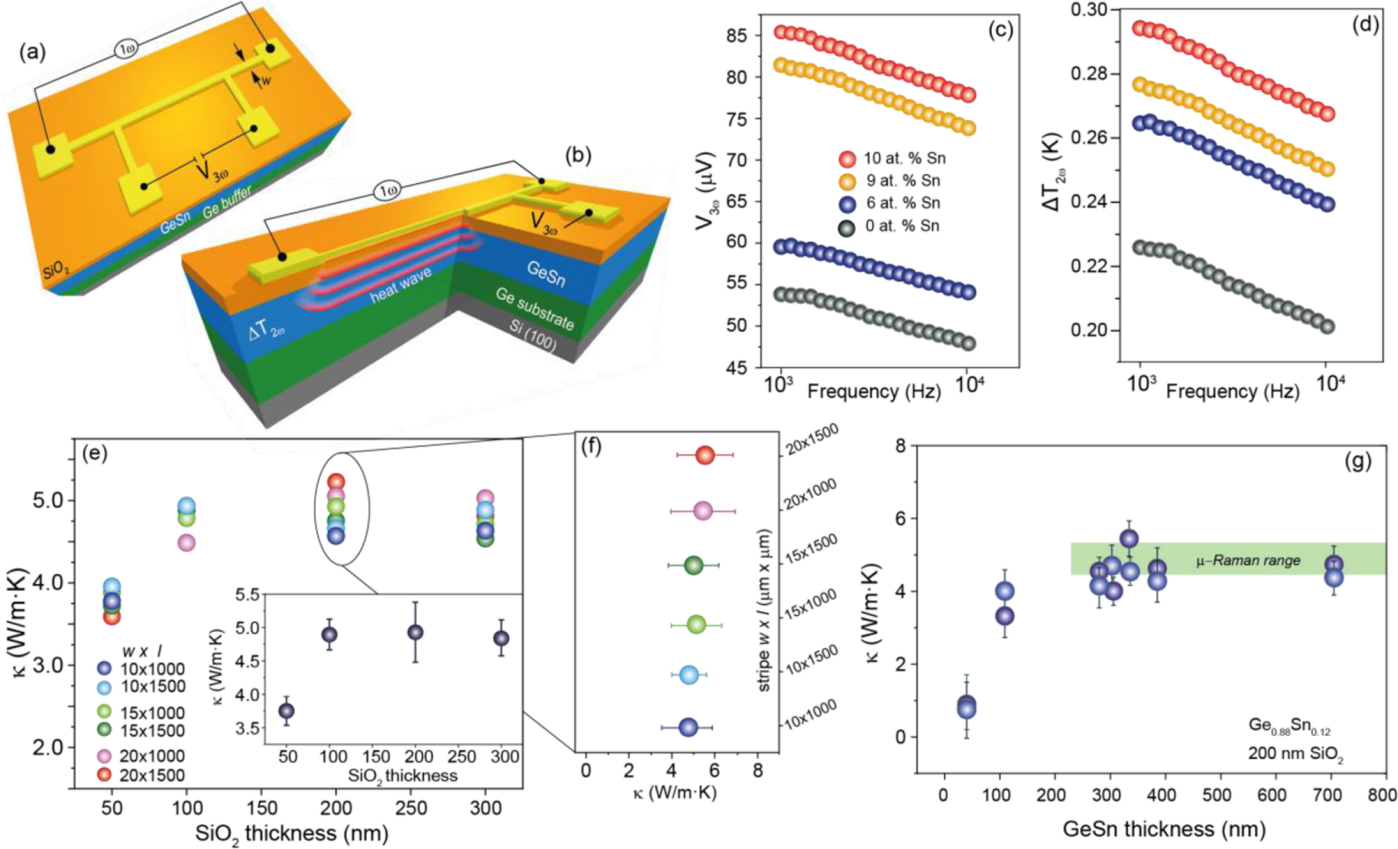
3ω methodology: (a) Schematic of a 3ω device and (b) heat wave propagation into the substrate. The sample is heated by driving a 1ω AC current through the metallic stripe. The temperature-dependent resistance of the stripe leads to a voltage drop *V*_3ω_, at the wire edges. (c) *V*_3ω_ voltage and (d) temperature variation Δ*T*_GeSn_ for GeSn layers with different Sn contents showing a typical linear dependence as a function of log(ω). (e) Thermal conductivity versus the SiO_2_ thickness for 350 nm Ge_0.88_Sn_0.12_ layers and different stripe geometries. The insets show the average thermal conductivity and (f) a zoom into κ for various stripe dimensions. (g) The average thermal conductivity for different Ge_0.88_Sn_0.12_ layer thicknesses. The Ge buffer is 300 nm thick in all cases except for the very thick 700 nm GeSn layer where the buffer is a very high crystallinity 1500 nm thick Ge layer. The values obtained by micro-Raman thermometry are marked in green.

The thermal lattice conductivity of GeSn epitaxial layers and its Sn dependence are measured using the differential 3ω-method.^34^ To implement this technique, metallic stripes are formed by standard lithography and lift-off processes on an insulating SiO_2_ layer deposited on the GeSn samples under investigation (see Figure 2a, b). The metallic stripes, composed of 50 nm Cr/250 nm Au, with different lengths (*l* = 1.0 and 1.5 mm) and widths (*w* = 10, 15, and 20 μm) are used to generate heat in the GeSn sample by Joule effect and, at the same time, as a local thermometer.

The 3ω-method exploits the temperature dependence of the stripe’s electrical resistance to measure the local lattice temperature. Joule heating takes place by driving an AC current of frequency ω through the metallic stripe. A heat wave of frequency 2ω is then induced into the sample. The heat dissipation, and consequently the local temperature variation Δ*T*_2ω_, at the sample top interface depends on the thermal conductivity and thickness of each layer in the sample stack. The local sample temperature variation is extracted from the measured voltage oscillation *V*_3ω_ at frequency 3ω, arising due to frequency mixing of the driving current and the resistivity, which follows the temperature oscillations. The lattice thermal conductivity of the GeSn layer is then deduced by fitting this temperature variation as detailed below. To this aim, the stripe temperature coefficient of resistance (TCR), α, has been preliminary measured in the 275–295 K temperature range, obtaining a value of α = (2.24–3.01 ± 0.05) × 10^–3^ K^–1^ as measured in two different laboratories.

The 3ω device design consists of a stripe with a width much larger than the epilayer thickness, allowing a 1D model description of the heat transport in this region and the usual cylindrical heat diffusion in the substrate.^34,35^ In this theoretical framework, the Δ*T*_2ω_ in a multilayer structure is related to the measured *V*_3ω_ and *V*_1ω_ signals via the thermal conductivity κ_*n*_ and layer thickness *t*_*n*_ of each *n*-th layer in the heterostructure (here the Ge buffer and the GeSn epilayer) by the following relation:
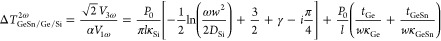
1awhere *P*_0_ is the total electrical input power over the stripe, γ is the Euler constant, κ_Si_ = 160 W/(m·K), and *D*_Si_ = 8 × 10^–5^ m^2^ s are the thermal conductivity and diffusivity of the Si substrate, respectively. Using a differential approach based on separate measurements on Ge/Si and GeSn/Ge/Si heterostructures and relying on the above equation, κ_GeSn_ is estimated using
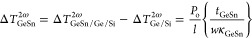
1bNote that eqs 1a, 1b hold provided that the heat flow in the epilayers can be considered as uniform, meaning that the conditions *t*_*n*_ ≪ *w* ≤ λ and *d* ≫ λ, where  is the thermal wavelength and *d* is the overall sample thickness, must be simultaneously satisfied, as is the case here for the frequency range of 1000–10000 Hz.

## Thermal Conductivity of GeSn Alloys

A set of typical *V*_3ω_ signal amplitudes for GeSn layers with different Sn content are presented in Figure 2c as a function of applied AC frequency. The data for the Ge/Si virtual substrate is used as a reference (0 at.% Sn). As predicted by the model, *V*_3ω_ amplitudes show a linear behavior as a function of log(ω) in the frequency region 1000 Hz < ω < 10000 Hz. The corresponding temperature oscillation amplitudes Δ*T*_2ω_ induced in the GeSn/Ge samples are shown in Figure 2d. It is apparent that the temperature of GeSn epilayers increases with the Sn content, pointing to a significantly lower heat dissipation through the GeSn material compared to the Ge reference.

To put our estimation of κ_GeSn_ on solid ground, different preliminary measurements were performed. First, the impact of the SiO_2_ insulating layer thickness on the predicted value of κ_GeSn_ is investigated. To this aim, a set of devices was fabricated from the same Ge_0.88_Sn_0.12_/Ge heterostructure wafer, with different thicknesses of the SiO_2_ electrical insulating layer (see Figure 1a, b). The κ_GeSn_ values, obtained from eq 1b are shown in Figure 2e. As observed, the extracted value of κ_*GeSn*_ is unaffected for an oxide thickness larger than 100 nm, while a lower thermal conductivity is obtained for the 50 nm SiO_2_ sample. This is in full agreement with literature reports ref. (35) and is attributed to an anomalously large SiO_2_ interface thermal resistance. For all further experiments reported here, the SiO_2_ thickness was chosen to be 200 nm. The data shown in Figure 2e, f also highlight that upon variation of the lateral width of the stripe *w* as well as the length *l*, stable values of κ_GeSn_ are obtained.

The thermal conductivity of Ge_0.88_Sn_0.12_ alloy is constant for layers with thicknesses in the range of 100–700 nm, for which the κ values are in agreement with previous Raman thermometry^9^ data (green area in Figure 2g). This layer thicknesses are above the critical thickness for strain relaxation, and the Ge_0.88_Sn_0.12_ layers are then highly strain relaxed. At a thickness of 50 nm, the Ge_0.88_Sn_0.12_ layers are pseudomorphic grown (lattice matched) on the Ge buffer layer and, as a consequence, under a very large biaxially compressive strain of about −1.6%, which may contribute to a lower thermal lattice conductivity. In addition, Khatami et al.^10^ theoretically predicted a decrease of the thermal conductivity with the GeSn layer thickness due to a reduction of the phonon mean free path.

**Figure 3:**
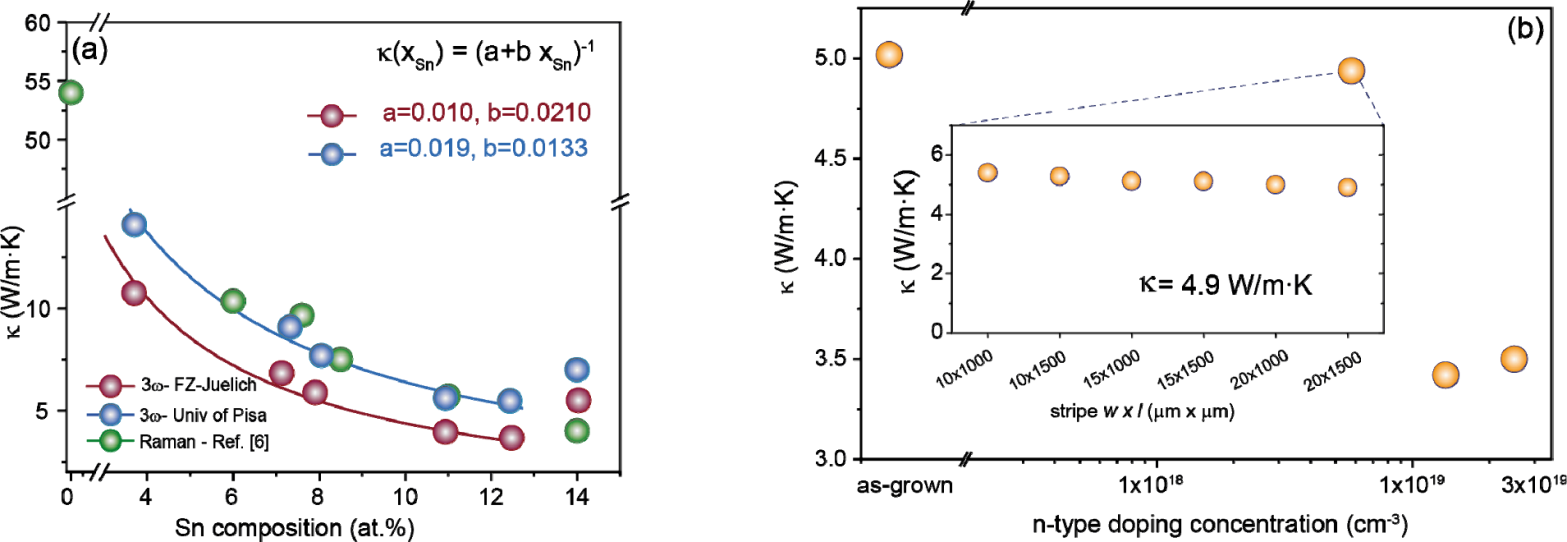
Thermal conductivity of GeSn alloys. (a) The dependence on Sn composition is obtained in two independent laboratories using the 3ω-method and by means of μ-Raman thermometry. The full lines are empirical fits using the equation *k*(*x*) = 1/(*a* + *bx*). The a and b coefficients for every case are given in the figure. (b) Lattice thermal conductivity of Ge_0.88_Sn_0.12_ layers as a function of n-type doping concentration. A slight decrease in κ is observed toward 1 × 10^19^ cm^–3^ doping level followed by a strong decrease after that. The inset shows the measurements obtained for different device stripe geometries.

The lattice thermal conductivity extracted using the 3ω-method for a set of GeSn samples with Sn content varying between 4 and 14 at.% is plotted in Figure 3. The two sets of data (blue and red points) represent the same devices measured in two independent laboratories. Each data point was obtained by averaging the results from six different devices. These results will be discussed in-depth later, and here we just point to the clear and consistent decrease of κ_GeSn_ with the Sn content.

The electrical 3ω-method data are compared with literature reported values^9^ measured using an optical method, μ-Raman thermometry (green dots in Figure 3a). The thermal conductivity values of GeSn alloys obtained by both electrical and optical methods are in good agreement, offering clear evidence of a significant decrease in thermal conductivity by alloying Ge with Sn. The trend is commonly observed in binary alloy materials, e.g., SiGe,^36^ and is ascribed to large phonon scattering rates induced by the alloy disorder, resulting in a “U-shaped” form for κ_SiGe_ versus Ge content, with a large plateau in the central Ge concentration region.

Differently from pure random SiGe alloys, the 3ω-method indicates slightly increased κ_*GeSn*_ values for Sn content over 12 at.%, which deviates from the trend discussed above. This effect is part of the peculiarities of GeSn alloys and is likely related to the atomic short-range ordering (SRO) at increased Sn concentration. The SRO was first observed in SiGeSn alloys^37^ and recently both theoretically and experimentally in GeSn alloys.^38,39^ Two types of SROs coexist in SiGeSn alloys, as predicted by DFT calculations: E-SRO (Enhanced-SRO) and R-SRO (Regular-SRO), with a strong impact on the electronic band structure.^38^ For GeSn alloys, there is only one type of SRO, namely regular SRO, that is shown to effectively increase the thermal conductivity of GeSn alloys.^40^

**Figure 4:**
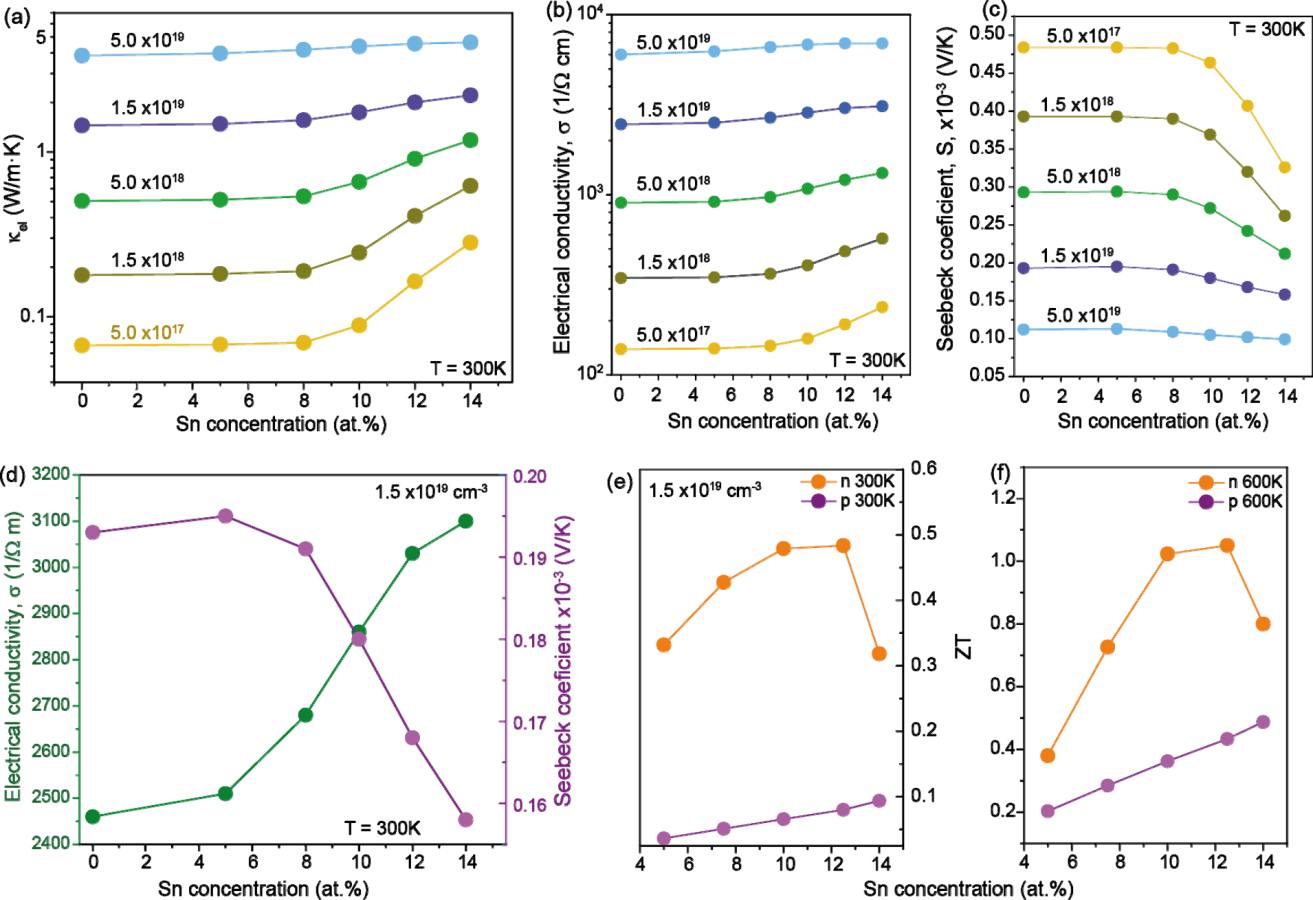
TE parameter modeling. (a) Electronic thermal conductivity κ_el_, (b) electrical conductivity σ, and (c) the Seebeck coefficient *S*, as a function of Sn content for different n-type doping concentrations at 300 K. (d) The charge transport coefficients for different Sn contents for n-type doping of 1.5 × 10^19^ cm^–3^, indicating the opposite behavior of σ and *S*. (e, f) *ZT* at a carrier density of 1.5 × 10^19^ cm^–3^ versus Sn composition, for p-type and n-type GeSn alloys at temperatures of (e) 300 K and (f) 600 K.

The rather low measured values of thermal lattice conductivity indicate the great potential of the GeSn system for low-heat thermoelectric applications. As already stated above, the measured thermal conductivity is essentially the lattice contribution, since for unintentional doping levels <5 × 10^17^ cm^–3^ the calculated electron thermal conductivity is at least 1 order of magnitude lower than the lattice conductivity. However, in view of TE applications, both the thermal part κ_GeSn_, as well as the electrical part *σS*^2^ of the figure of merit *ZT* = *σS*^2^*/κ* must be optimized. In this context, a set of GeSn:P layers has been grown (Figure 1f), and only their measured total thermal conductivity, shown in Figure 3b, is in the scope of this manuscript. A slight increase in thermal conductivity, by ∼15%, is measured up to carrier densities of 1 × 10^19^ cm^–3^ (inset Figure 3b), above which a significant decrease of κ_GeSn_ is observed. This looks like being in contradiction with the theory that suggests an increase of κ_el_ with increasing doping (see Figure 4a). Actually, this behavior is related to an increase of the phonon scattering, as has been reported for different material systems.^41,42^ Indeed, in some large bandgap III–V semiconductors the room-temperature thermal conductivity decreases with the logarithm of the carrier density.^42^ In group IV at large doping levels, the activation rate of the dopants varies between 50% and 70%,^43,44^ leading to a large number of impurities that are not active electrically and do not contribute to the expected increase of the electron lattice thermal conductivity. The activated dopants, according to recent experiments, may also enhance the electron–phonon scattering,^45^ leading to an overall lower thermal conductivity. The above results indicate that an optimum doping concentration has to be found between the negative influence on the thermal conductivity and the positive influence on the electrical properties of the GeSn alloys.

## Thermoelectric Parameters Modeling

Combining the measured values of thermal conductivity with theoretical predictions of the electronic transport properties, the figure of merit *ZT* and the optimum Sn content range for p- and n-type-doped Ge_1–*x*_Sn_*x*_ alloys can be estimated. To this aim numerical simulations based on the Boltzmann transport equation,^46,47,36^ exploring the parameter space spanned by doping, Sn concentration, and lattice temperature were performed. To capture the impact of the intervalley scattering process occurring in the conduction band of this quasi-direct material, the carrier occupancy of the lowest minimum in the conduction band and of the other Brillouin-zone minima close in energy, as well as the related intervalley scattering events have been taken into account. The scattering mechanisms included were (i) electron–phonon coupling with acoustic and optical lattice excitations; (ii) the Coulomb scattering induced by charged impurities; (iii) scattering on neutral, point-like defects; and (iv) alloy disorder scattering.

The core of the simulation results is presented in Figure 4, where for n- and p-type GeSn alloys the Sn content dependences of the electron thermal conductivity κ_el_, electrical conductivity σ, and Seebeck coefficient *S*, are shown for different doping levels and lattice temperatures. Figure 4b, c indicates that in n-type GeSn σ and *S* feature opposite trends upon varying the Sn content and the doping concentration, resulting in a maximum *ZT*. Indeed, *ZT* values initially increase with Sn content, driven by the improvement in σ, but with further increase in Sn content σ boost flattens and *S* decreases, leading to the peak in *ZT* observed in Figure 4d for a fixed and illustrative value of carrier density. This behavior is driven by the relative charge carrier occupation of the L and Γ conduction valleys. In the high-Sn content region, due to the small DOS of the Γ-valley, the Fermi level energy shifts up into the conduction band, therefore reducing the Seebeck coefficient which overcompensates the increase in electrical conductivity. On the other hand, in the low-Sn content range where the bottom of the conduction band is at the L point, it is just the dynamics of σ which dominates the positive *ZT* trend. This results in the peak-like trend in *ZT* observed in Figure 4d.

This effect is not observed in p-type materials, where the hole occupancy at the top of the broad valence band maximum depends only slightly on the doping concentration. In this case, *ZT* increases monotonically as a function of the Sn content, driven by the reduction of the lattice conductivity, as can be observed in Figures 4e, f for a p-GeSn atom with the same carrier concentration and at two different temperatures. The plots indicate that large *ZT* values can be obtained for GeSn layers with Sn contents of 10–12 at.% at 1–1.5 × 10^19^ cm^–3^ doping, and such layers can be epitaxially grown with the required high crystalline quality,^17,18^ making the group IV alloys ready for testing for TE device applications.

## Conclusions

The thermal conductivity of GeSn alloys was measured for a large range of material parameters such as stoichiometry, layer thickness, and doping. The data show that Sn incorporation leads to bulk thermal conductivities ∼30 times lower than that of Si and 10 times lower than that of Ge. The GeSn alloys with Sn content of 12 at.% with thicknesses between 100 and 700 nm show a constant thermal conductivity, as low as 5 W/(m·K). These data clearly indicate that the κ reduction obtained by alloying Ge and Sn boosts the TE performance of GeSn alloys above that of other CMOS and MEMS compatible materials, namely SiGe alloys or tensile strained Ge when operated at low temperatures. Large *ZT* values for n-type GeSn samples can be obtained by optimizing the phosphorus concentration to epitaxially achievable values. The presented data indicate that GeSn alloys represent a very viable alternative to other IV–VI semiconductors, traditionally used for TE applications, due to their nontoxicity, abundance, and compatibility with the CMOS standard. Furthermore, this compatibility adds another major advantage: scalability and miniaturization, which is not achievable by leveraging conventional thermoelectric materials.

## References

[ref1] Ling-Chin J.; Bao H.; Ma Z.; Taylor W.; Paul Roskilly A. State-of-the-Art Technologies on Low-Grade Heat Recovery and Utilization in Industry. In Energy Conversion - Current Technologies and Future Trends; IntechOpen, 2019. 10.5772/intechopen.78701

[ref2] Luberti M.; Gowans R.; Finn P.; Santori G. An Estimate of the Ultralow Waste Heat Available in the European Union. Energy 2022, 238, 121967 10.1016/j.energy.2021.121967.

[ref3] Dong B.; Shi Q.; Yang Y.; Wen F.; Zhang Z.; Lee C. Technology Evolution from Self-Powered Sensors to AIoT Enabled Smart Homes. Nano Energy 2021, 79, 105414 10.1016/j.nanoen.2020.105414.

[ref4] Basu R.; Singh A. High Temperature Si–Ge Alloy towards Thermoelectric Applications: A Comprehensive Review. Mater. Today Phys. 2021, 21, 100468 10.1016/j.mtphys.2021.100468.

[ref5] He R.; Heyn W.; Thiel F.; Pérez N.; Damm C.; Pohl D.; Rellinghaus B.; Reimann C.; Beier M.; Friedrich J.; Zhu H.; Ren Z.; Nielsch K.; Schierning G. Thermoelectric Properties of Silicon and Recycled Silicon Sawing Waste. J. Mater. 2019, 5 (1), 15–33. 10.1016/j.jmat.2018.11.004.

[ref6] Xie J.; Chizmeshya A. V. G.; Tolle J.; D’Costa V. R.; Menendez J.; Kouvetakis J. Synthesis, Stability Range, and Fundamental Properties of Si–Ge–Sn Semiconductors Grown Directly on Si(100) and Ge(100) Platforms. Chem. Mater. 2010, 22 (12), 3779–3789. 10.1021/cm100915q.

[ref7] von den Driesch N.; Stange D.; Wirths S.; Rainko D.; Povstugar I.; Savenko A.; Breuer U.; Geiger R.; Sigg H.; Ikonic Z.; Hartmann J.; Grützmacher D.; Mantl S.; Buca D. SiGeSn Ternaries for Efficient Group IV Heterostructure Light Emitters. Small 2017, 13 (16), 1603321 10.1002/smll.201603321. 28160408

[ref8] Von Den Driesch N.; Stange D.; Wirths S.; Mussler G.; Holländer B.; Ikonic Z.; Hartmann J. M.; Stoica T.; Mantl S.; Grützmacher D.; Buca D. Direct Bandgap Group IV Epitaxy on Si for Laser Applications. Chem. Mater. 2015, 27 (13), 4693–4702. 10.1021/acs.chemmater.5b01327.

[ref9] Spirito D.; von den Driesch N.; Manganelli C. L.; Zoellner M. H.; Corley-Wiciak A. A.; Ikonic Z.; Stoica T.; Grützmacher D.; Buca D.; Capellini G. Thermoelectric Efficiency of Epitaxial GeSn Alloys for Integrated Si-Based Applications: Assessing the Lattice Thermal Conductivity by Raman Thermometry. ACS Appl. Energy Mater. 2021, 4 (7), 7385–7392. 10.1021/acsaem.1c01576.

[ref10] Khatami S. N.; Aksamija Z. Lattice Thermal Conductivity of the Binary and Ternary Group-IV Alloys Si-Sn, Ge-Sn, and Si-Ge-Sn. Phys. Rev. Appl. 2016, 6 (1), 1–11. 10.1103/PhysRevApplied.6.014015.

[ref11] Bao S.; Zhu W.; Yu Y.; Liang L.; Deng Y. Wearable Thermoelectric Generator with Cooling-Enhanced Electrode Design for High-Efficient Human Body Heat Harvesting. ACS Appl. Eng. Mater. 2023, 1 (1), 660–668. 10.1021/acsaenm.2c00167.

[ref12] Kim C. S.; Yang H. M.; Lee J.; Lee G. S.; Choi H.; Kim Y. J.; Lim S. H.; Cho S. H.; Cho B. J. Self-Powered Wearable Electrocardiography Using a Wearable Thermoelectric Power Generator. ACS Energy Lett. 2018, 3 (3), 501–507. 10.1021/acsenergylett.7b01237.

[ref13] Tang J.; Gao B.; Lin S.; Wang X.; Zhang X.; Xiong F.; Li W.; Chen Y.; Pei Y. Manipulation of Solubility and Interstitial Defects for Improving Thermoelectric SnTe Alloys. ACS Energy Lett. 2018, 3 (8), 1969–1974. 10.1021/acsenergylett.8b01098.

[ref14] Roychowdhury S.; Biswas R. K.; Dutta M.; Pati S. K.; Biswas K. Phonon Localization and Entropy-Driven Point Defects Lead to Ultralow Thermal Conductivity and Enhanced Thermoelectric Performance in (SnTe)1–2 x(SnSe)x(SnS)X. ACS Energy Lett. 2019, 4 (7), 1658–1662. 10.1021/acsenergylett.9b01093.

[ref15] Liu W. Di; Wang D. Z.; Liu Q.; Zhou W.; Shao Z.; Chen Z. G. High-Performance GeTe-Based Thermoelectrics: From Materials to Devices. Adv. Energy Mater. 2020, 10 (19), 2000367 10.1002/aenm.202000367.

[ref16] Kang Y.; Xu S.; Han K.; Kong E. Y. J.; Song Z.; Luo S.; Kumar A.; Wang C.; Fan W.; Liang G.; Gong X. Ge0.95Sn0.05Gate-All-Around p-Channel Metal-Oxide-Semiconductor Field-Effect Transistors with Sub-3 Nm Nanowire Width. Nano Lett. 2021, 21 (13), 5555–5563. 10.1021/acs.nanolett.1c00934. 34105972

[ref17] Liu M.; Junk Y.; Han Y.; Yang D.; Bae J. H.; Frauenrath M.; Hartmann J.-M.; Ikonic Z.; Bärwolf F.; Mai A.; Grützmacher D.; Knoch J.; Buca D.; Zhao Q.-T. Vertical GeSn Nanowire MOSFETs for CMOS beyond Silicon. Commun. Eng. 2023, 2 (1), 1–9. 10.1038/s44172-023-00059-2.

[ref18] Liu M.; Yang D.; Shkurmanov A.; Bae J. H.; Schlykow V.; Hartmann J.-M.; Ikonic Z.; Baerwolf F.; Costina I.; Mai A.; Knoch J.; Grützmacher D.; Buca D.; Zhao Q.-T. Epitaxial GeSn/Ge Vertical Nanowires for p-Type Field-Effect Transistors with Enhanced Performance. ACS Appl. Nano Mater. 2021, 4 (1), 94–101. 10.1021/acsanm.0c02368.

[ref19] Stange D.; Wirths S.; Geiger R.; Schulte-Braucks C.; Marzban B.; von den Driesch N.; Mussler G.; Zabel T.; Stoica T.; Hartmann J.-M. Optically Pumped GeSn Microdisk Lasers on Si. ACS Photonics 2016, 3 (7), 1279 10.1021/acsphotonics.6b00258.

[ref20] Stange D.; Von Den Driesch N.; Zabel T.; Armand-Pilon F.; Rainko D.; Marzban B.; Zaumseil P.; Hartmann J. M.; Ikonic Z.; Capellini G.; Mantl S.; Sigg H.; Witzens J.; Grützmacher D.; Buca D. GeSn/SiGeSn Heterostructure and Multi Quantum Well Lasers. ACS Photonics 2018, 5 (11), 4628–4636. 10.1021/acsphotonics.8b01116.

[ref21] Wirths S.; Geiger R.; von den Driesch N.; Mussler G.; Stoica T.; Mantl S.; Ikonic Z.; Luysberg M.; Chiussi S.; Hartmann J. M.; Sigg H.; Faist J.; Buca D.; Grützmacher D. Lasing in Direct-Bandgap GeSn Alloy Grown on Si. Nat. Photonics 2015, 9 (2), 88–92. 10.1038/nphoton.2014.321.

[ref22] Elbaz A.; Buca D.; von den Driesch N.; Pantzas K.; Patriarche G.; Zerounian N.; Herth E.; Checoury X.; Sauvage S.; Sagnes I.; Foti A.; Ossikovski R.; Hartmann J. M.; Boeuf F.; Ikonic Z.; Boucaud P.; Grützmacher D.; El Kurdi M. Ultra-Low-Threshold Continuous-Wave and Pulsed Lasing in Tensile-Strained GeSn Alloys. Nat. Photonics 2020, 14 (6), 375–382. 10.1038/s41566-020-0601-5.

[ref23] Marzban B.; Seidel L.; Liu T.; Wu K.; Kiyek V.; Zoellner M. H.; Ikonic Z.; Schulze J.; Grützmacher D.; Capellini G.; et al. Strain Engineered Electrically Pumped SiGeSn Microring Lasers on Si. ACS Photonics 2023, 10 (1), 217–224. 10.1021/acsphotonics.2c01508.

[ref24] Zhou Y.; Miao Y.; Ojo S.; Tran H.; Abernathy G.; Grant J. M.; Amoah S.; Salamo G.; Du W.; Liu J.; Margetis J.; Tolle J.; Zhang Y.; Sun G.; Soref R. A.; Li B.; Yu S.-Q. Electrically Injected GeSn Lasers on Si Operating up to 100 K. Optica 2020, 7 (8), 924 10.1364/OPTICA.395687.

[ref25] Talamas Simola E.; Kiyek V.; Ballabio A.; Schlykow V.; Frigerio J.; Zucchetti C.; De Iacovo A.; Colace L.; Yamamoto Y.; Capellini G.; et al. CMOS-Compatible Bias-Tunable Dual-Band Detector Based on GeSn/Ge/Si Coupled Photodiodes. ACS Photonics 2021, 8 (7), 2166–2173. 10.1021/acsphotonics.1c00617.

[ref26] Maeda S.; Ishiyama T.; Nishida T.; Ozawa T.; Saitoh N.; Yoshizawa N.; Suemasu T.; Toko K. High Thermoelectric Performance in Polycrystalline GeSiSn Ternary Alloy Thin Films. ACS Appl. Mater. Interfaces 2022, 14 (49), 54848–54854. 10.1021/acsami.2c14785. 36450141

[ref27] Uchida N.; Maeda T.; Lieten R. R.; Okajima S.; Ohishi Y.; Takase R.; Ishimaru M.; Locquet J. P. Carrier and Heat Transport Properties of Polycrystalline GeSn Films on SiO2. Appl. Phys. Lett. 2015, 107 (23), 232105 10.1063/1.4937386.

[ref28] Uchida N.; Hattori J.; Lieten R. R.; Ohishi Y.; Takase R.; Ishimaru M.; Fukuda K.; Maeda T.; Locquet J. P. Carrier and Heat Transport Properties of Poly-Crystalline GeSn Films for Thin-Film Transistor Applications. J. Appl. Phys. 2019, 126 (14), 145105 10.1063/1.5085470.

[ref29] Concepción O.; Søgaard N. B.; Bae J. H.; Yamamoto Y.; Tiedemann A. T.; Ikonic Z.; Capellini G.; Zhao Q. T.; Grützmacher D.; Buca D. Isothermal Heteroepitaxy of Ge1-XSnx Structures for Electronic and Photonic Applications. ACS Appl. Electron. Mater. 2023, 5, 2268 10.1021/acsaelm.3c00112. 37124237 PMC10134428

[ref30] Concepción O.; Søgaard N. B.; Bae J. H.; Yamamoto Y.; Tiedemann A. T.; Ikonic Z.; Capellini G.; Zhao Q. T.; Grützmacher D.; Buca D. Isothermal Heteroepitaxy of Ge1-XSnx Structures for Electronic and Photonic Applications. ACS Appl. Electron. Mater. 2023, 5 (4), 2268–2275. 10.1021/acsaelm.3c00112. 37124237 PMC10134428

[ref31] Rainko D.; Ikonic Z.; Vukmirović N.; Stange D.; von den Driesch N.; Grützmacher D.; Buca D. Investigation of Carrier Confinement in Direct Bandgap GeSn/SiGeSn 2D and 0D Heterostructures. Sci. Reports 2018 81 2018, 8 (1), 1–13. 10.1038/s41598-018-33820-1. PMC6197271 30348982

[ref32] Moontragoon P.; Ikonić Z.; Harrison P. Band Structure Calculations of Si–Ge–Sn Alloys: Achieving Direct Band Gap Materials. Semicond. Sci. Technol. 2007, 22 (7), 742–748. 10.1088/0268-1242/22/7/012.

[ref33] Graziosi P.; Li Z.; Neophytou N. ElecTra Code: Full-Band Electronic Transport Properties of Materials. Comput. Phys. Commun. 2023, 287, 108670 10.1016/j.cpc.2023.108670.

[ref34] Cahill D. G. Thermal Conductivity Measurement from 30 to 750 K: The 3ω Method. Rev. Sci. Instrum. 1990, 61 (2), 802–808. 10.1063/1.1141498.

[ref35] Lee S. M.; Cahill D. G. Heat Transport in Thin Dielectric Films. J. Appl. Phys. 1997, 81 (6), 2590–2595. 10.1063/1.363923.

[ref36] Li Y.; Wang G.; Akbari-Saatlu M.; Procek M.; Radamson H. H. Si and SiGe Nanowire for Micro-Thermoelectric Generator: A Review of the Current State of the Art. Front. Mater. 2021, 8 (March), 1–24. 10.3389/fmats.2021.611078.

[ref37] Mukherjee S.; Kodali N.; Isheim D.; Wirths S.; Hartmann J. M.; Buca D.; Seidman D. N.; Moutanabbir O. Short-Range Atomic Ordering in Nonequilibrium Silicon-Germanium-Tin Semiconductors. Phys. Rev. B 2017, 95 (16), 161402 10.1103/PhysRevB.95.161402.

[ref38] Jin X.; Chen S.; Li T. Coexistence of Two Types of Short-Range Order in Si–Ge–Sn Medium-Entropy Alloys. Commun. Mater. 2022, 3 (1), 1–9. 10.1038/s43246-022-00289-5.

[ref39] Corley-Wiciak A. A.; Chen S.; Concepción O.; Zoellner M. H.; Grützmacher D.; Buca D.; Li T.; Capellini G.; Spirito D. Local Alloy Order in a Ge1–xSnx/Ge Epitaxial Layer. Phys. Rev. Appl. 2023, 20 (2), 024021 10.1103/PhysRevApplied.20.024021.

[ref40] Cao B.; Chen S.; Jin X.; Liu J.; Li T. Short-Range Order in GeSn Alloy. ACS Appl. Mater. Interfaces 2020, 12 (51), 57245–57253. 10.1021/acsami.0c18483. 33306349

[ref41] Asheghi M.; Kurabayashi K.; Kasnavi R.; Goodson K. E. Thermal Conduction in Doped Single-Crystal Silicon Films. J. Appl. Phys. 2002, 91 (8), 5079–5088. 10.1063/1.1458057.

[ref42] Zou J.; Kotchetkov D.; Balandin A. A.; Florescu D. I.; Pollak F. H. Thermal Conductivity of GaN Films: Effects of Impurities and Dislocations. J. Appl. Phys. 2002, 92 (5), 2534–2539. 10.1063/1.1497704.

[ref43] Lanzerath F.; Buca D.; Trinkaus H.; Goryll M.; Mantl S.; Knoch J.; Breuer U.; Skorupa W.; Ghyselen B. Boron Activation and Diffusion in Silicon and Strained Silicon-on-Insulator by Rapid Thermal and Flash Lamp Annealings. J. Appl. Phys. 2008, 104 (4), 044908 10.1063/1.2968462.

[ref44] Minamisava R. A.; Buca D.; Heiermann W.; Lanzerath F.; Mantl S.; Skorupa W.; Hartmann J.-M.; Ghyselen B.; Kernevez N.; Breuer U. Flash Lamp Activation of N- and p-Type Dopants in Strained and Unstrained SOI and HOI. ECS Trans. 2009, 19, 79–86. 10.1149/1.3118933.

[ref45] Pan Z.; Yang L.; Tao Y.; Zhu Y.; Xu Y. Q.; Mao Z.; Li D. Net Negative Contributions of Free Electrons to the Thermal Conductivity of NbSe3nanowires. Phys. Chem. Chem. Phys. 2020, 22 (37), 21131–21138. 10.1039/D0CP03484C. 32959836

[ref46] Graziosi P.; Kumarasinghe C.; Neophytou N. Material Descriptors for the Discovery of Efficient Thermoelectrics. ACS Appl. Energy Mater. 2020, 3 (6), 5913–5926. 10.1021/acsaem.0c00825.

[ref47] Graziosi P.; Neophytou N. Ultra-High Thermoelectric Power Factors in Narrow Gap Materials with Asymmetric Bands. J. Phys. Chem. C 2020, 124 (34), 18462–18473. 10.1021/acs.jpcc.0c05457.

